# Underreporting of high-risk water and sanitation practices undermines progress on global targets

**DOI:** 10.1371/journal.pone.0176272

**Published:** 2017-05-10

**Authors:** Sridhar Vedachalam, Luke H. MacDonald, Solomon Shiferaw, Assefa Seme, Kellogg J. Schwab, On behalf of PMA2020 investigators

**Affiliations:** 1 Johns Hopkins Water Institute, Johns Hopkins Bloomberg School of Public Health, Baltimore, MD 21205 United States of America; 2 School of Public Health, Addis Abbaba University, Addis Abbaba, Ethiopia; Leibniz Institute for Prvention Research and Epidemiology BIPS, GERMANY

## Abstract

Water and sanitation indicators under the Millennium Development Goals failed to capture high-risk practices undertaken on a regular basis. In conjunction with local partners, fourteen rounds of household surveys using mobile phones with a customized open-source application were conducted across nine study geographies in Asia and Africa. In addition to the main water and sanitation facilities, interviewees (n = 245,054) identified all water and sanitation options regularly used for at least one season of the year. Unimproved water consumption and open defecation were targeted as high-risk practices. We defined underreporting as the difference between the regular and main use of high-risk practices. Our estimates of high-risk practices as the main option matched the widely accepted Demographic and Health Surveys (DHS) estimates within the 95% confidence interval. However, estimates of these practices as a regular option was far higher than the DHS estimates. Across the nine geographies, median underreporting of unimproved water use was 5.5%, with a range of 0.5% to 13.9%. Median underreporting of open defecation was much higher at 9.9%, with a range of 2.7% to 11.5%. This resulted in an underreported population of 25 million regularly consuming unimproved water and 50 million regularly practicing open defecation. Further examination of data from Ethiopia suggested that location and socio-economic factors were significant drivers of underreporting. Current global monitoring relies on a framework that considers the availability and use of a single option to meet drinking water and sanitation needs. Our analysis demonstrates the use of multiple options and widespread underreporting of high-risk practices. Policies based on current monitoring data, therefore, fail to consider the range of challenges and solutions to meeting water and sanitation needs, and result in an inflated sense of progress. Mobile surveys offer a cost-effective and innovative platform to rapidly and repeatedly monitor critical development metrics.

## Introduction

The Millennium Development Goals (MDGs) and ongoing Sustainable Development Goals (SDGs) are arguably the most important global development policy tools used to set national priorities for Water, Sanitation, and Hygiene (WASH) [1]. The MDGs were established by the United Nations to address a series of interconnected issues including poverty, health, sanitation and education, ending in 2015 [2]. Subsequently, the Sustainable Development Goals were adopted in September 2015. The WHO/UNICEF’s Joint Monitoring Programme (JMP) tracked progress toward MDG targets via the drinking water and sanitation ladders (see Box 1). These same indicators will be used to track SDG progress. JMP indicators on progress towards safe water and improved sanitation frequently justify development aid [3,4]; set benchmarks that drive national policy [1]; and are prominently cited in scholarly work [5,6]. Therefore, these metrics must be as accurate as possible. While the JMP’s methods are statistically sound, JMP indicators have several notable shortcomings inherent in their design that lead to inaccuracies. These must be addressed to make substantive progress that improves lives on the ground, not just in reports.

**Box 1**

Drinking water sources

Piped water on premises*

- Piped water to dwelling, plot or yard

Other improved sources*

- Public taps or standpipes
- Tubewell/borewell
- Protected dug well
- Protected spring
- Rainwater

Unimproved sources

- Unprotected dug wells
- Unprotected springs
- Cart with a small tank/drum
- Tanker truck
- Bottled and sachet water

Surface water

- River, lake, pond or canal water

Sanitation facilities

Improved (unshared)^#^

- Flush/pour flush to piped sewer, septic or pit latrine
- Ventilated improved pit (VIP) latrine
- Pit latrine with slab
- Composting toilet

Improved (shared)

- Improved facilities shared between two or more households

Unimproved

- Pit latrine without slab
- Hanging latrine
- Bucket latrine

Open defecation

- Field, forest, bush, or open body of water

*Both piped water and ‘other improved’ sources are together classified as improved sources. ^#^Unlike the case of drinking water, only unshared improved facilities are counted toward improved sanitation. Source: WHO/UNICEF [7].

JMP indicators suffer from three critical shortcomings. The first is that they track household infrastructure classes without regard to service quality, in part due to cost constraints [8,9]–improved water sources are not necessarily safe [5,10], and flush toilets connected to sewers often empty waste directly into the environment [11]. A second shortcoming is that JMP indicators capture the presence or absence of a facility without assessing usage rates. Questionnaires developed by the USAID-led Demographic and Health Surveys (DHS), which form the basis for JMP estimates, ask respondents to self-identify their main drinking water source or main sanitation facility. The indicators do not address the reliability of these sources, despite evidence that water sources in many cases are unreliable [12] or that infrequent sanitation services such as emptying of pit latrines present an important barrier to widespread use [13]. Higher rates of household toilets do not necessarily result in favorable sanitation outcomes at the city-level [14].

A third critical shortcoming is that JMP indicators do not capture multiple household water sources or sanitation facilities because they only track a single main option. JMP indicators rest on the central assumption that this main option exists, but many studies show that households rarely rely on a single option to meet their needs [15,16]. Households often use other regular sources to augment the main source, a concept sometimes called source-switching [17]. Variations among members within a single household even arise, especially for open defecation [15]. Therefore, indicators that solely rely on a main option can substantially underreport high-risk practices such as drinking water from an unimproved source or practicing open defecation.

The regular use of secondary options raises the possibility that individuals expose themselves to pathogens via their own high-risk practices or through contact with other household members who undertake high-risk practices, as shown by Spears et al. [18], although data on this issue are limited. Depending on the frequency of source-switching, size of the household, etc., such individuals may be exposed to pathogens as often as members of households that use high-risk options as the main option. Thus, progress on indicators based on the main option may not reflect meaningful improvements in protecting public health.

In addition to accurate indicators, there is a need for more frequent monitoring of progress against amibitous WASH targets such as the elimination of open defecation for everyone, everywhere. Consider, for example, that the 2015 JMP update reports that 29% of Ethiopia’s 94.10 million people practice open defecation [19]. An average of 1.8 million people must adopt an alternative sanitation practice each year until 2030 to meet the SDGs, with even more adopters needed if considering population growth, and yet more still if considering the underreporting discussed above.

Traditional field surveys such as those led by national governments and the gold-standard DHS are very expensive, so they are conducted infrequently or spaced apart several years. Owing to their omnibus nature, they typically attempt to collect a large volume of information, and therefore face necessary constraints on the number of questions. Such constraints often result in the failure to capture the complexity of certain household characteristics or behavioral attributes. The restricted number of questions coupled with the time gap between surveys is not suited for policy adjustments needed to stay on track for attaining SDG targets.

Mobile phone technologies and the use of resident enumerators present one way to meet this monitoring challenge by making surveys inexpensive and rapid. Mobile surveys are increasingly being used to assess a range of population outcomes [20–22], with results similar to and often more reliable than paper surveys [23,24]. The Field Level Operations Watch (FLOW), a water point mapping software developed by the Water and Sanitation Program of the World Bank, mapped 7,500 water points across rural Liberia in less than a month [25]. The Sanitation Hygiene Infant Nutrition Efficacy (SHINE) trial in Zimbabwe to estimate cluster-specific water and sanitation access using an open-source geospatial software is another recent example [26]. However, the WASH sector has seen fewer large-scale digital data collection initatives that span several countries.

The digital collection survey presented in this article, Performance Monitoring and Accountability 2020 (PMA2020, http://pma2020.org), is a large-scale mobile phone based monitoring program led by Johns Hopkins University (JHU) in collaboration with local research institutions in each study country [27]. PMA2020 surveys are modular, allowing for inclusion of newer topics while consistently monitoring core metrics. Currently, the PMA2020 surveys include family planning and WASH modules, and surveys are conducted at 6-month intervals in households and health care facilities.

Using PMA2020 data from nine geographies across eight countries, this article addresses underreporting of unimproved water consumption and open defecation, which are targeted as high-risk practices. Underreporting is defined as the difference between the prevalence of regular and main high-risk practices. This article seeks to document and quantify underreporting at, to our knowledge, an unprecedented scale. The overarching aim is to demonstrate that high-level reports which show consistent progress towards development targets do not necessarily reflect the day-to-day experiences of residents of developing countries.

The specific aims of this article are to: (1) demonstrate and quantify the occurrence of underreporting in key water and sanitation metrics used globally to measure public health progress, (2) identify socio-demographic characteristics of underreported individuals in one study geography, and (3) explore policy recommendations to address this critical shortcoming. To meet goal 2, this article presents regression modeling results from Ethiopia, where our analysis discovered comparatively high levels of underreporting. Ethiopia is a priority country for USAID-led WASH interventions and is one of the top receipients of WASH aid from the U.S. government [28,29].

## Methods

Results in this article come from the PMA2020 study countries listed in Table 1. Most are national surveys but some are subnational as indicated. In each country, a local research institution manages training, data collection, and dissemination, while JHU provides technical support. The survey was approved by ethical institutional review boards in each country and at JHU. A list of IRBs that approved the PMA2020 protocol is included at the end of this article.

**Table 1:** Descriptors of the data used in the analysis.

Country	Code	Scope	Rounds	Total respondents
**Burkina Faso**	BF	National	1, 2	4,166
**Congo, Dem. Rep.**	CDK	Kinshasa	1	21,596
**Ethiopia**	ET	National	1, 2, 3	86,243
**Ghana**	GH	National	1, 2	30,483
**Indonesia**	ID	National	1	45,006
**Niger**	NEN	Niamey	1	6,032
**Nigeria**	NGK	Kaduna state	1	11,401
**Nigeria**	NGL	Lagos state	1	3,597
**Uganda**	UG	National	1, 2	36,530
**Grand Total**				**245,054**

Notes: All countries are denoted by a standard two-alphabet code, followed by a third alphabet in cases where the surveys were restricted to a limited region of the country. Total respondents include only complete surveys and *dejure* population.

### Sample selection

PMA2020 uses a two-tiered study design. Working alongside each national statistics office, PMA2020 draws clusters of households according to an urban-rural stratification scheme with the number of clusters proportional to the population distribution, which is the first study tier. Each cluster (also called Enumeration Area or EA) is designed to have an average of 200 households. A subset of households is selected for interview, generally around 40 households per EA depending on the desired sample size, which is the second tier.

To generate the pool of households in each cluster, female resident enumerators (REs) complete a mapping and listing of all households within cluster boundaries. Households are not necessarily families, but rather a group of one or more people who share a common pot of food. Household may dwell within one or more structures, and multiple households may reside within a single structure. Sample weights derive from the initial probability of selection, adjusted for the measured pool of all households within a cluster.

### Household interviews

A common household questionnaire, translated into local languages, was administered to all respondents. REs visited each household up to three times to identify a competent respondent. The survey included basic demographics of all household members, such as sex, age, relationship to the household head, and assets such as television, radio, and bicycle. Ownership of assets was used to calculate wealth scores according to Filmer and Pritchett [30] and Rutstein and Johnson [31]. The resulting wealth scores can take negative or positive values, and the position of a household on the wealth spectrum was an indicator of its wealth relative to other households.

Drinking water results included responses to the question “Which of the following water sources does your household use on a regular basis for any part of the year for any purpose?” REs read aloud a list of water sources matching those used in the country’s DHS. Sources used several times per week during at least one season of the year met the criteria for regular use. If more than one source was provided, households were asked to identify a main source for drinking and a main source for cooking or washing (all other purposes), using DHS question wording. Due to limitations on interview time and to limit recall bias, additional questions to quantify the use of regular sources in relation to the main source were not included in the questionnaire.

Respondents specified household uses of each regular water source, including drinking. The household also estimated the perceived reliability of this source during the time of year it was expected to be available as either (i) always available, (ii) predictably intermittent, or (iii) unpredictable. Households also estimated the time taken to go to each source, collect water, and return.

Sanitation related content presented in this article focuses on open defecation. Households first identified all sanitation facilities and then the main sanitation facility using country DHS categories and question wording for the main facility. Later in the questionnaire, respondents were asked “How many people within your household regularly use the bush/field at home or at work?” to directly estimate regular practice of open defecation irrespective of whether open defecation was reported earlier. Use of open defecation several times per week during at least one season of the year met the criteria for regular use. Although there was no equivalent question to assess the reliability of the sanitation source, respondents indicated the frequency of using a particular source through a categorical four-point scale.

### Mobile platform

PMA2020 uses a version of the open-source Open Data Kit (ODK) Collect app for Android phones to collect repsonses to single- or multiple-select questions, dates, and numerical results. REs may toggle between languages in order to conduct interviews in local languages.

PMA2020 ODK questionnaires have embedded skip patterns, so appropriate questions automatically appear based on previous responses. Custom constraints prevent nonsensical responses, such as selecting “none of the above” with another choice. Warning screens flag unusual responses so the RE can confirm accurate data entry. The length of survey time varies, but routinely falls between 20 and 30 minutes in length. When complete, REs submit interview data to a secure server using the cellular data network or a Wi-Fi network. Central staff download aggregate data for cleaning, removal of identifiers, and analysis.

### Data management

For this article, de-identified data from several rounds conducted 6 months apart were aggregated, where possible, to generate a single dataset for each country (Table 1). The resulting dataset is an average of independent random samples drawn according to the sampling strategy described earlier. Incomplete responses were dropped. All results are de-jure population, i.e. for usual household members based on the roster. The analysis was conducted using Stata v14.1 [32].

### Model, variables and hypothesis

For each study country, estimates for the main and regular use of high-risk practices along with their respective 95% CI were calculated. Estimates from the most recent DHS were included for comparison, with confidence intervals calculated according to the stratification scheme and sample weights on de-identified raw data downloaded with permission from DHS website (www.dhsprogram.com).

The difference between the regular and main PMA2020 estimates is classified here as underreporting of the high-risk practice. In practical terms, it means that a user with a non-high-risk source as the main option switches to a high-risk source as one of their regular options. Taking the case of Ethiopia, the disparity in the urban-rural use of and underreporting of unimproved drinking water (UW) and open defecation (OD) were highlighted. UW and OD use and underreporting by wealth was displayed using locally weighted polynomial regression. Binary logistic (logit) regression models were built to study in detail the socio-demographic and structural factors associated with underreporting of UW and OD in Ethiopia, separately. The binary dependent variable in each model was the underreported status of a usual household member, and was scored ‘yes’ or 1 if the respondent used a non-high-risk source as their main option, but supplemented that with a high-risk source as a regular option. The dependent variable was scored 0 if the main and regular options were both non-high-risk.

The independent explanatory variables included socio-economic characteristics, structural factors, and a geographic regional control. Socio-economic variables included a dummy for rural location of the household, normalized wealth score, and household size. Structural factors for drinking water included number of water sources, reliability of the main source, and time to collect water from the main source. Structural factors for sanitation include the reported frequency of using the main facility. Number of sanitation sources was considered a poor indicator of underreporting and thus not included in the model since only six percent of the respondents in Ethiopia reported using using more than one source, but many more reported using OD for their sanitation needs via a separate question that only focused on OD behavior. Estimates are presented as odds ratios, with standard errors robust to clustering by Enumeration Areas (EAs) in one of the models.

## Results

**Fig 1:**
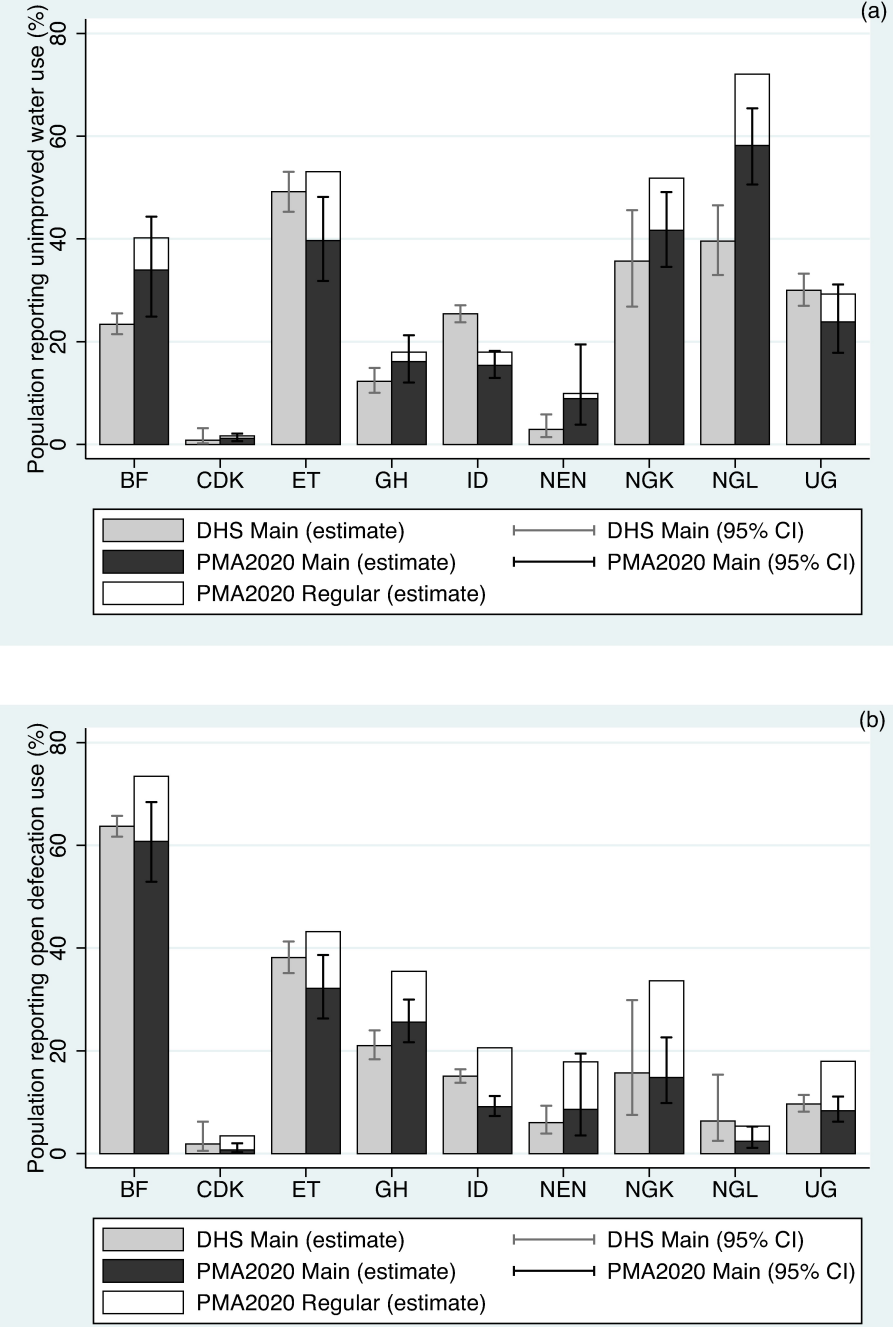
Underreporting of high-risk practices. Main and regular estimates of (a) unimproved water, and (b) open defecation.

Fig 1 shows underreporting of UW (Fig 1A) and OD (Fig 1B). Underreporting of high risk practices was found in all study geographies for both UW and OD. PMA2020 estimates for high-risk practices as the main option matched DHS estimates within the 95% confidence interval in all study geographies, except Indonesia and Nigeria (Lagos; NGL) for UW and Indonesia for OD.

The magnitude of underreporting varied across the study areas. Underreported UW ranged from 0.51 percent in DR Congo (Kinshasa; CDK) to 13.87 percent in NGL. Similarly, underreported OD ranged from 2.74 percent in CDK to 18.83 percent in NGL. Overall, the underreporting of OD (median: 9.86 percent; Uganda) was much higher than that of unimproved water use (median: 5.45 percent; Ghana).

Based on these nine geographies, we identified an underreported population of 25 million regularly consuming unimproved drinking water, and 50 million regularly practicing open defecation (Tables 2 and 3). Though we observed wide variation in the underreported population across the study regions, these numbers were approximately 5.5 and 11 percent of the total population in these regions.

**Table 2:** Estimates of main, regular and underreported use of unimproved water.

Country Code	Population (million)	Unimproved water			
		Main source (percent)	Regular source (percent)	Underreported (percent)	Underreported (million)
BF	16.93	33.92	40.22	6.30	1.07
CDK	10.67	1.18	1.69	0.51	0.05
ET	94.10	39.70	53.06	13.36	12.57
GH	25.91	16.14	17.94	1.80	0.47
ID	249.86	15.39	17.98	2.59	6.47
NEN	1.03	8.93	9.92	0.99	0.01
NGK	7.56	41.65	51.80	10.15	0.77
NGL	11.27	58.18	72.05	13.87	1.56
UG	37.58	23.84	29.29	5.45	2.05
Total	454.91				25.02

Note: The national population estimates were obtained from The World Bank (2015). Regional population estimates for CDK, NEN and NGK/NGL were obtained from UN-HABITAT [33], Institut National De La Statistique [34] and the National Population Commission [35], respectively. All estimates, except for CDK (2015) and NEN (2012), are for the year 2013. CDK estimate is a projection for the year 2015; the last census was conducted in 1984. NGK and NGL estimates are based on the national annual growth rate of 3.08% between the 2006 Census and the 2013 World Bank estimate [36].

**Table 3:** Estimates of main, regular and underreported use of open defecation.

Country Code	Population (million)	Open defecation			
		Main source (percent)	Regular source (percent)	Underreported (percent)	Underreported (million)
BF	16.93	60.77	73.45	12.68	2.15
CDK	10.67	0.73	3.47	2.74	0.29
ET	94.10	32.14	43.22	11.08	10.43
GH	25.91	25.61	35.47	9.86	2.55
ID	249.86	9.13	20.60	11.47	28.66
NEN	1.03	8.59	17.86	9.27	0.12
NGK	7.56	14.82	33.65	18.83	1.42
NGL	11.27	2.43	5.36	2.93	0.33
UG	37.58	8.35	17.95	9.60	3.61
Total	454.91				49.56

Note: The national population estimates were obtained from The World Bank (2015). Regional population estimates for CDK, NEN and NGK/NGL were obtained from UN-HABITAT [33], Institut National De La Statistique [34] and the National Population Commission [35], respectively. All estimates, except for CDK (2015) and NEN (2012), are for the year 2013. CDK estimate is a projection for the year 2015; the last census was conducted in 1984. NGK and NGL estimates are based on the national annual growth rate of 3.08% between the 2006 Census and the 2013 World Bank estimate [36].

**Fig 2:**
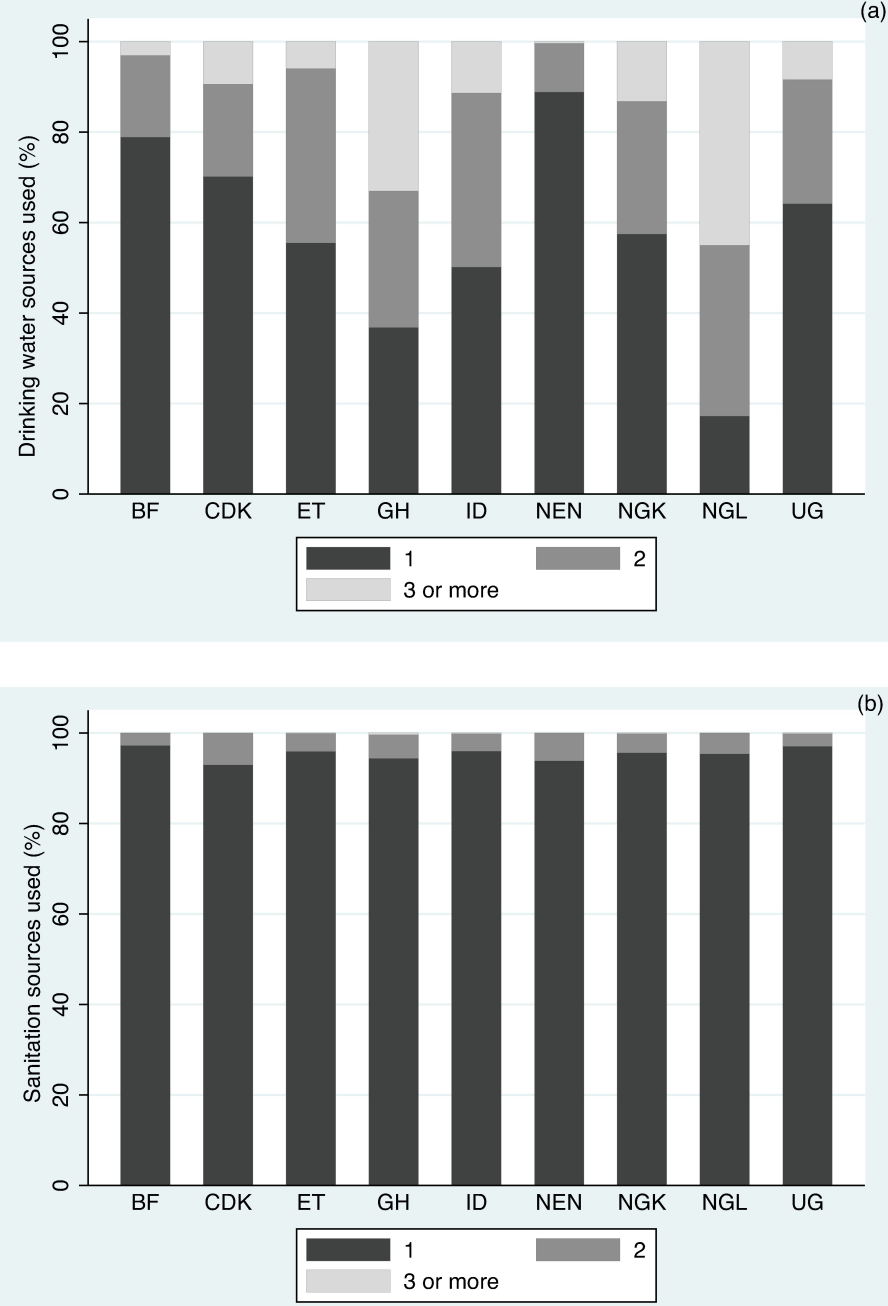
Availability of water and sanitation options. Number of sources used by respondents for (a) drinking water, and (b) sanitation.

Next, we demonstrated the shortcoming of assuming a single water and sanitation source by way of data from each of our study geographies (Fig 2). More than 95 percent of the respondents in our nine study regions claimed to use just a single sanitation facility when asked to identify all facilities (mean 95.37%; S.D. 1.34%) (Fig 2B). When the regular practice of OD was assessed through a direct question, however, the practice was found to be widespread (Table 3). In contrast, less than 60 percent of the respondents reported that their household relied on just one water source (mean 57.68%, S.D. 20.42%) (Fig 2A).

**Fig 3:**
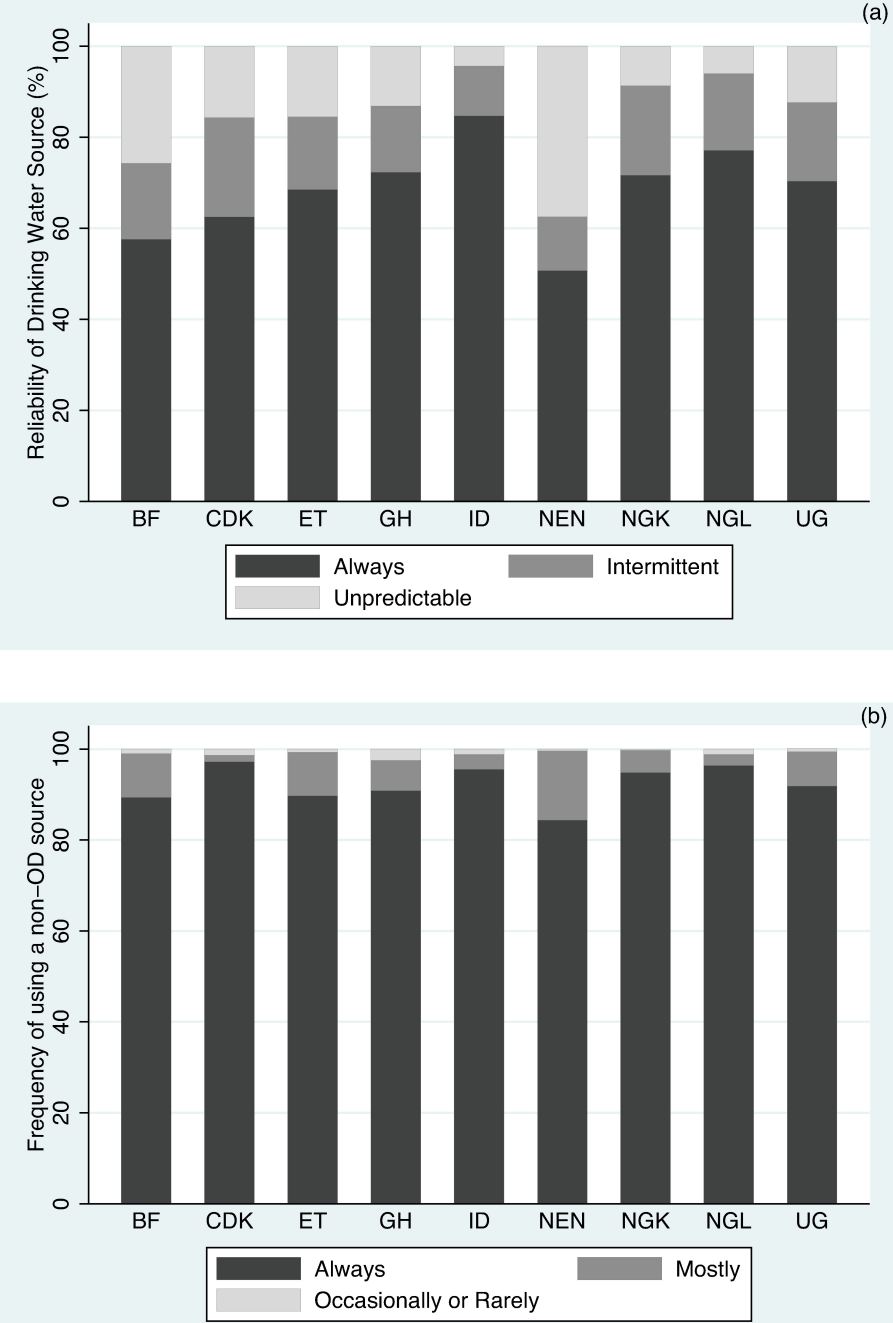
Likelihood of using non-high-risk practices. (a) Reliability of the main water option, when the source is improved. (b) Frequency of using a non-OD source as the main sanitation option.

Among the population that belonged to households with an improved main drinking water source, Fig 3A shows the reported reliability of that source. Those with a source that was ‘always available’ ranged from 50.71% (NEN) to 84.70% (ID), with a median 70.36% (UG). The rest of the population reported either predictably intermittent or unpredicatable supplies. It is conceivable that users experiencing an intermittent or unpredictable improved water source may occasionally turn to a more reliable unimproved source. In the case of sanitation, the frequency of ‘always’ using a non-OD sanitation option as the main source ranged from 84.40% (NEN) to 97.22% (CDK), with a median of 91.85% (UG) (Fig 3B). Although a large fraction of users reported ‘always’ using a non-OD source, we found a much higher use of OD on a regular basis. In all but two study geographies, underreported OD users were greater than those self-reporting that they ‘mostly’, ‘occasionally’ or ‘rarely’ used a non-OD source, which suggests that some users who claimed to ‘always’ use a non-OD source might be openly defecating.

To further explore relationships between the use of high-risk WASH practices and socio-economic characteristics such as location and wealth, we further analyzed data from Ethiopia. Ethiopia recorded a large underreported population, and is a USAID priority WASH country [28,29]. Ethiopia is the largest population among PMA2020 geographies except Indonesia and had the largest multi-round pooled sample size. Although results from Ethiopia are not generalizable to other study geographies, they yield powerful insights on HH decisions on water and sanitation.

In Ethiopia, the proportion of rural residents undertaking high-risk practices as the main option was much greater than urban residents (Tables 4 and 5). However, this disparity between urban and rural residents dropped when considering all regular options. The total number of underreported users of UW and OD were spread roughly evenly between urban and rural residents, but due to wider disparity in the main use of UW and OD across urban and rural areas, the relative underreporting in urban areas expressed as a proportion of the main users of high-risk practices was much higher than in rural areas.

**Table 4:** Main, regular and underreported use of unimproved water sources in Ethiopia across urban and rural locations.

Location	Unimproved water users (percent)			Underreporting ratio
	Main	Regular	Underreported	
Urban	4.13	15.33	11.20	2.71
Rural	46.71	60.48	13.77	0.29
Total	39.70	53.06	13.36	0.34

Note: Underreported users are obtained by subtracting the main users from regular users. Underreporting ratio is the ratio of underreported users to the main users.

**Table 5:** Main, regular and underreported use of open defecation in Ethiopia across urban and rural locations.

Location	Open defecation users (percent)			Underreporting ratio
	Main	Regular	Underreported	
Urban	8.56	17.45	8.89	1.04
Rural	36.80	48.30	11.50	0.31
Total	32.14	43.22	11.08	0.34

Note: Underreported users are obtained by subtracting the main users from regular users. Underreporting ratio is the ratio of underreported users to the main users.

**Fig 4:**
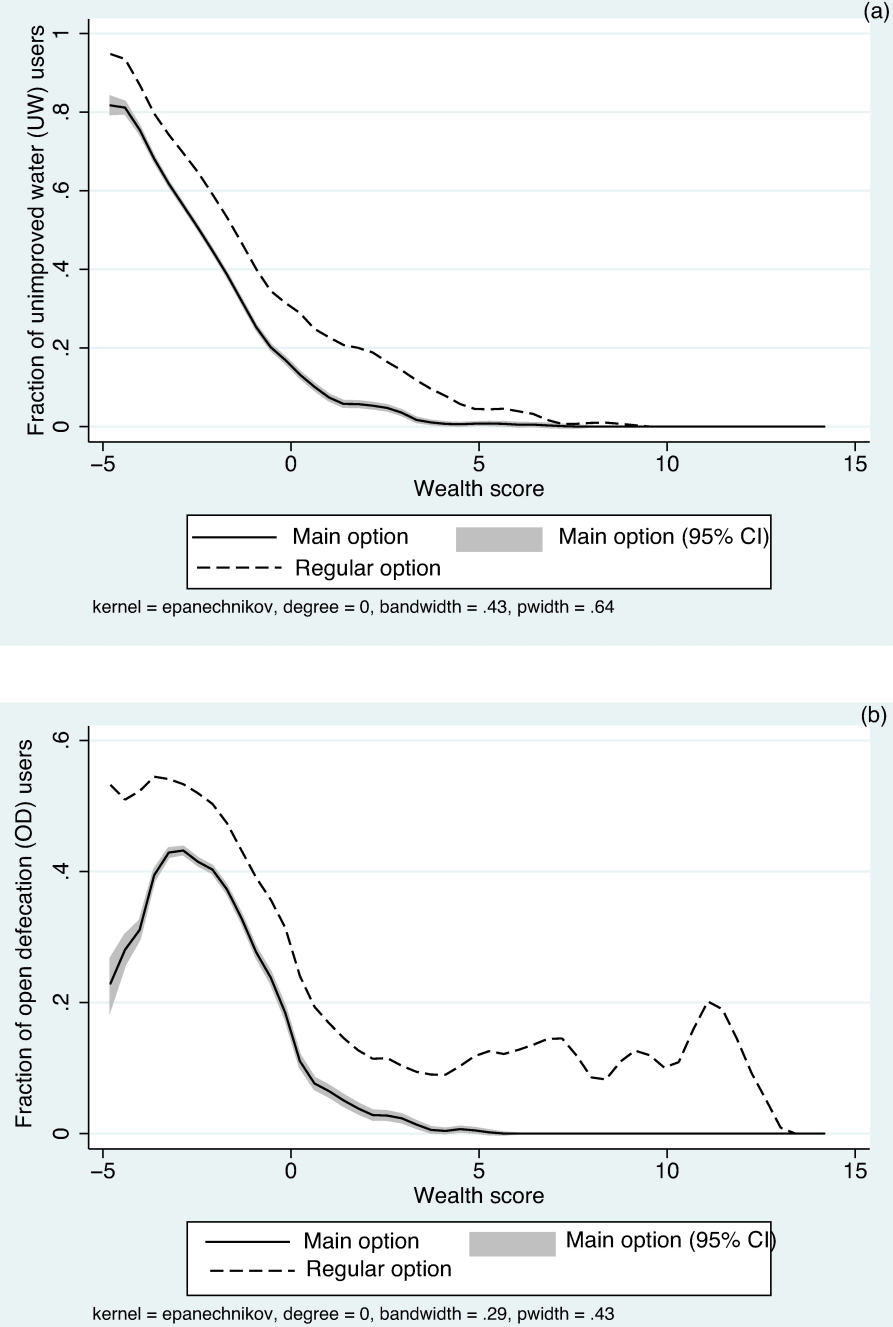
High-risk practice as a function of wealth. Local polynomial smoothing function showing (a) unimproved water use, and (b) open defecation, over wealth score in Ethiopia.

Fig 4 shows the locally weighted polynomial regression of UW and OD against wealth score. UW use, both as the main and regular option, declined with greater wealth in Ethiopia (Fig 4A). Underreporting, seen as the vertical distance between the two curves at any given wealth score, stayed relatively constant among lower wealth levels and declined at high wealth levels in the case of UW. OD as main or regular practice also displayed an overall negative association with wealth (Fig 4B). However, underreporting was high at both extremes of the wealth spectrum and relatively constant in between.

### Underreporting in Ethiopia

Next, we investigated structural, demographic and socio-economic factors associated with underreporting of high-risk WASH practices in Ethiopia. Table 6 describes the variable definitions and summary statistics for this data set. Tables 7 and 8 show logistic regression models where the underreported use of UW and OD are dependent variables, and we sequentially build each model with the addition of independent variables. Prelim (1) was our basic model, and contained variables that explain socio-economic setup of the household (location, wealth score and household size). Prelim (2) added a regional control (region) and structural attributes relevant to water or sanitation. Final (3) subsequently added characteristics associated with the household head, and was our fully expanded model. We then analyzed the Final (3) model using standard errors robust to clustering to arrive at the Clustered (4) model.

**Table 6:** Variable definitions and summary statistics.

Variable	Description	Min	Max	Mean	St. Dev.	Median
DW_underrep	Underreported user of unimproved drinking water	0	1	0.13	0.34	0
OD_underrep	Underreported user of open defecation	0	1	0.14	0.35	0
Rural	Location of the household; 1 if rural, 0 otherwise	0	1	0.84	0.37	1
Wealth score	Index of household wealth derived from ownership of select assets	-4.80	14.18	-1.42	2.16	-2.01
Household Size	Number of household members	1	19	5.63	2.22	6
Water Sources	Number of water sources; observations with just one water source excluded	2	5	2.15	0.38	2
Water Reliability						
1. Always		0	1	0.76	0.43	1
2. Predictably intermittent		0	1	0.13	0.34	0
3. Unpredictable		0	1	0.11	0.31	0
Collection Time	Time to collect water daily, round-trip (hours)	0	14	0.60	1.14	0.25
Sanitation frequency						
1. Always		0	1	0.91	0.28	1
2. Mostly, occasionally or rarely		0	1	0.09	0.28	0
Education	Level of education of the household head	0	4	0.07	0.37	0
Age	Age of the household head (divided by 10)	0	12	4.25	1.68	4
Woman	Sex of the household head; 1 if woman, 0 otherwise	0	1	0.18	0.38	0

**Table 7:** Logistic regression results for underreporting of unimproved water in Ethiopia.

Variable	Prelim (1)	Prelim (2)	Final (3)	Clustered (4)
Rural	0.909* [0.822, 1.006]	1.386*** [1.234, 1.555]	1.419*** [1.252, 1.608]	1.419 [0.659, 3.054]
Wealth score	0.740*** [0.721, 0.760]	0.786*** [0.765, 0.808]	0.786*** [0.764, 0.809]	0.786*** [0.662, 0.934]
Household size	1.041*** [1.027, 1.055]	0.965*** [0.949, 0.982]	0.956** [0.937, 0.976]	0.956 [0.900, 1.016]
Water sources		2.461*** [2.238, 2.706]	2.540*** [2.300, 2.803]	2.540*** [1.597, 4.039]
Water reliability				
1. Always		-	-	-
2. Predictably intermittent		0.720*** [0.646, 0.802]	0.746*** [0.664, 0.837]	0.746 [0.428, 1.299]
3. Unpredictable		1.375*** [1.216, 1.555]	1.254*** [1.100, 1.430]	1.254 [0.711, 2.213]
Water collection		1.138*** [1.111, 1.165]	1.138*** [1.109, 1.168]	1.138 [0.949, 1.364]
Education			0.991 [0.886, 1.110]	0.991 [0.748, 1.313]
Age			0.949*** [0.923, 0.975]	0.949 [0.891, 1.010]
Woman			0.925 [0.821, 1.042]	0.925 [0.685, 1.250]
Constant	0.191*** [0.164, 0.222]	0.0152*** [0.009, 0.023]	0.0220*** [0.013, 0.035]	0.0220*** [0.002, 0.230]
Region control		Yes	Yes	Yes
Clustering				Yes
N	63,436	27,174	23,674	23,674
Pseudo R^2^	0.049	0.092	0.096	0.096

Notes: Parameter estimates are odds ratios, with 95% confidence intervals in brackets.

*p < 0.10

**p < 0.05

***p < 0.01.

**Table 8:** Logistic regression results for underreporting of open defecation in Ethiopia.

Variable	Prelim (1)	Prelim (2)	Final (3)	Clustered (4)
Rural	1.586*** [1.454, 1.729]	1.790*** [1.631, 1.965]	1.796*** [1.621, 1.990]	1.796** [1.095, 2.947]
Wealth score	0.902*** [0.887, 0.918]	0.866*** [0.849, 0.883]	0.865*** [0.847, 0.883]	0.865** [0.773, 0.967]
Household size	1.028*** [1.015, 1.041]	1.028*** [1.014, 1.042]	1.042*** [1.026, 1.059]	1.042 [0.986, 1.102]
Sanitation frequency Always		-	-	-
Mostly, Ocassionally or Rarely		2.358*** [2.166, 2.566]	2.228*** [2.032, 2.442]	2.228*** [1.668, 2.974]
Education			1.106*** [1.027, 1.192]	1.106 [0.943, 1.298]
Age			0.963*** [0.943, 0.985]	0.963 [0.913, 1.016]
Woman			0.948 [0.860, 1.045]	0.948 [0.756, 1.189]
Constant	0.081*** [0.071, 0.094]	0.121*** [0.102, 0.143]	0.131*** [0.107, 0.161]	0.131*** [0.043, 0.396]
Region control		Yes	Yes	Yes
Clustering				Yes
N	62,939	62,144	53,464	53,464
Pseudo R^2^	0.022	0.056	0.056	0.056

Notes: Parameter estimates are odds ratios, with 95% confidence intervals in brackets.

**p < 0.05

***p < 0.01.

When evaluating the Final (3) model, underreporting of UW was less likely to be observed among wealthy (OR = 0.786; 95% CI [0.662–0.934]) and larger households (0.956; 0.937–0.976), but more likely in rural households (1.419; 1.252–1.608) and when residents had access to multiple sources (2.540; 2.300–2.803) (Table 7). In comparison to an always-reliable main source, UW underreporting was likely to be higher in the case of an unpredictable main source (1.254; 1.100–1.430), whereas having a predictably intermittent source decreased the odds of underreporting (0.746; 0.664–0.837). Longer collection times were associated with underreporting (1.138; 1.109–1.168). Underreporting was less likely when household head was older (0.949; 0.923–0.975). Sex and education of the household head were not significant drivers of underreporting for drinking water. Once the effect of clustering by EAs was factored in the Clustered (4) model, only wealth score and access to multiple sources were significant.

In the case of OD, underreporting as observed in the Final (3) model was less likely among wealthy households (OR = 0.865, 95% CI [0.847–0.883]) but more likely in rural areas (1.796; 1.621–1.990) and among larger households (1.042; 1.026–1.059). Underreporting was more likely among users who reported using their main sanitation facility ‘mostly’, ‘ocassionally’ or ‘rarely’, as opposed to ‘always’ using them (2.228; 2.032–2.442). Underreporting was more likely when the household head was highly educated (1.106; 1.027–1.192) and less likely when the household head was older (0.963; 0.943–0.985). Sex of the household head was not a significant driver of underreporting. Once the effect of clustering by EAs was factored in the Clustered (4) model, variables such as HH size and characteristics of the HH head dropped in significance, and only living in a rural area, wealth score and frequency of sanitation facility use were significantly associated with underreporting.

## Discussion

Existing indicators underreport the use of high-risk practices in all nine study geographies because they rely on one main water and sanitation option. PMA2020 estimates closely tracked the DHS estimates, except in case of ID (for UW and OD) and NGL (for UW only). The discrepancy in UW results for ID and NGL are likely due to higher reporting of packaged water use (refill water in ID and sachet in NGL, respectively) in our surveys as compared to DHS. We don’t have an explanation for the OD results in ID. We found that household use of multiple water sources was widespread, and we uncovered large populations regularly practicing OD despite having access to a sanitation facility. Across nine study geographies, the cumulative underreported population using high-risk practices was 5.5% for UW and 11% for OD. This group of underreported users is currently not considered when setting national or global targets.

Of the two high-risk practices considered here, underreporting of open defecation was more common than unimproved water use in all but two study areas. The SDGs’ focus on elimination of open defecation suggests that it is the highest sanitation priority [37]. Although this study uncovered high underreporting of OD, it is possible that the true rate of OD may be even higher because of the social stigma associated with OD reporting during interviews [38].

The regression models developed for Ethiopia yield insights on socio-economic and structural factors associated with underreporting of high-risk practices. Residents of rural, less wealthy households in Ethiopia and those with a younger household head were likely to switch to high-risk practices on a regular basis. Access to multiple water sources, and the quality of the main water and sanitation option also played critical roles in this behavior. Longer water collection times and infrequent use of the main sanitation facility were likely to result in the switch to a high-risk regular practice. Having a female household head did not affect the switching behavior. Regional differences were observed in both UW and OD models; those results are not shown or discussed here.

Household size played a distinctly different role in each model. Underreporting or source-switching was less likely in larger households for UW use, but more likely in larger households for OD use. The effect of source reliability on UW underreporting was a bit surprising. As compared to having an always-reliable main option, an unpredictable option resulted in higher rates of source-switching, whereas a predictably intermittent option led to a lower likelihood of source-switching. This might be possible in a scenario where respondents view the unpredictable option as the least reliable, but the predictably intermittent option is treated at par or considered more valuable than the always-available option due to the superiority (aesthetic or otherwise) of that source. This result is less clear and needs further investigation.

The use of cluster-robust standard errors identified fewer factors as drivers of underreporting once structural differences across EAs were accounted for. Wealth was the only consistently significant factor among both UW and OD models. The Tiebout sorting theory would suggest that residents choose their communities based on the offerings and services provided by the community, and would move (“vote with their feet”) to a different community if the present one doesn’t satisfy their needs [39]. However, this is not practical in several cases. Our results suggest that even in resource-challenged communities, wealthy HHs have access to more reliable sources as their main options and are thus less likely to be underreported and than their poorer counterparts.

The explanation for the role played by factors such as HH size and water source reliability on underreporting may be unclear, but the overarching pattern is easy to identify: poor, disadvantaged Ethiopian households use multiple water and sanitation options to meet their needs. In Ethiopia, more work is needed to understand the mechanisms and decision-making behind households’ reliance on multiple WASH options and their switch to high-risk practices. Likewise, more work is needed to identify whether factors that contribute to underreporting in Ethiopia extend to other countries.

This work presents a new way to monitor progress on the challenge of securing safe water and sanitation across the world. Using a mobile phone monitoring platform at the country-level, we illustrated that PMA2020 estimates provided results comparable to established surveys such as the DHS on much shorter timelines. Due to the modular nature of PMA2020 surveys, new questions can be rapidly and inexpensively added to develop a deeper understanding every 6 months rather than every few years, a leap forward that allows tracking of the ambitious SDGs and other WASH interventions, as needed.

Using new metrics, PMA2020 consistently found underreporting of high-risk WASH practices, illustrating a serious flaw in existing JMP indicators. The JMP is the gold-standard in monitoring health and development outcomes around the world and influences agenda-setting at the national and global levels. The SDGs have set ambitious new targets, including the elimination of open defecation by 2030, and our analysis showed that the challenge to meeting these targets is likely to be greater than that anticipated using standard metrics. Recent changes to the JMP indicators, such as the inclusion of source reliability in the decision to classify a source as “improved”, are a step in the right direction, yet fall short due to their continued reliance on the main option alone and ignoring the prevalent use of multiple sources.

Based on the results presented in this study, the authors recommend that changes be made to the current monitoring protocol used to track global targets. The presence of multiple water and sanitation options, particularly the use of regular options, need to be incorporated to accurately assess the WASH landscape. Measuring the reliability and use frequency of the main option can be additional standard metrics that explain when and why users switch to high-risk practices. These changes in WASH monitoring will more accurately assess the realities of everyday life in a complex world, and have wide-ranging impacts on global and national policy-making.

## Abbreviations

CI: Confidence interval
DHS: Demographic and Health Surveys
JMP: Joint Monitoring Programme
MDG: Millennium Development Goals
OR: Odds Ratio
OD: Open defecation
PMA2020: Performance Monitoring and Accountability 2020
SDG: Sustainable Development Goals
UNICEF: United Nations Children’s Fund
USAID: United States Agency for International Development
UW: Unimproved water
WASH: Water, Sanitation and Hygiene
WHO: World Health Organization
